# Supplementary material for: A Putative D-Arabinono-1,4-lactone Oxidase, MoAlo1, Is Required for Fungal Growth, Conidiogenesis, and Pathogenicity in Magnaporthe oryzae
Source: J Fungi (Basel). 2022 Jan 11;8(1):72. doi: 10.3390/jof8010072 (PMC8782026; doi:10.3390/jof8010072)
Supplement: Supplementary file 1 [file jof-08-00072-s001.zip › jof-1521373-supplementary.pdf]

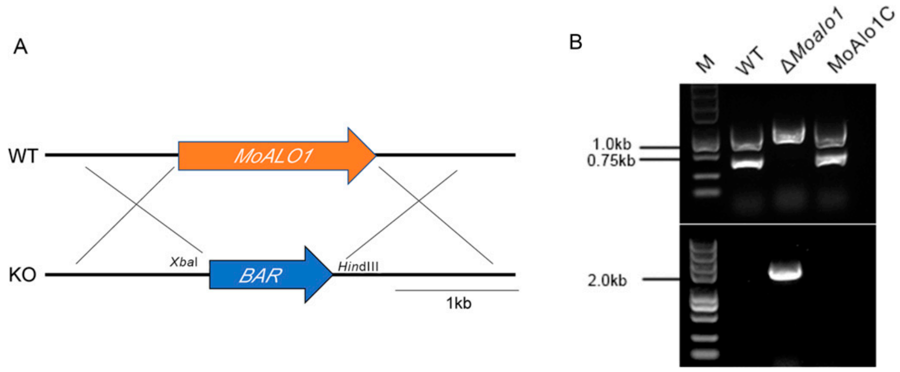

Figure S1. Gene deletion of *MoALO1* in *M. oryza*e. (A) Gene replacement of *MoALO1* locus. WT: wild type; KO: knockout. (B) The null mutant was verified by double PCR. *β-tubulin* gene (1.0 kb) were used as a positive control. A distinct band (~0.5 kb) was produced in the wild type Guy11 and the complementary strain MoAlo1C, but Δ*Moalo1* mutant did not. The null mutant was further verified by PCR, the Δ*Moalo1* mutant produced a ~2.0 kb band indicating the target gene deletion cassette.
